# Insights from teaching artificial intelligence to medical students in Canada

**DOI:** 10.1038/s43856-022-00125-4

**Published:** 2022-06-03

**Authors:** Ricky Hu, Kevin Y. Fan, Prashant Pandey, Zoe Hu, Olivia Yau, Minnie Teng, Patrick Wang, Toni Li, Mishal Ashraf, Rohit Singla

**Affiliations:** 1 School of Medicine, Queen’s University, Kingston, ON Canada; 2 School of Biomedical Engineering, The University of British Columbia, Vancouver, BC Canada; 3 Department of Radiation Oncology, The University of Toronto, Toronto, ON Canada; 4 Faculty of Medicine, The University of British Columbia, Vancouver, BC Canada

**Keywords:** Health care, Computational biology and bioinformatics

## Abstract

Hu et al. describe their experiences running a training course for medical students about applying artificial intelligence to medical practice. They also provide recommendations for future training programs.

## Teaser

Clinical artificial intelligence (AI) applications are rapidly developing but existing medical school curricula provide limited teaching covering this area. Here we describe an AI training curriculum we developed and delivered to Canadian medical undergraduates and provide recommendations for future training.

## Introduction

Artificial intelligence (AI) in medicine can potentially create workplace efficiencies and aid in clinical decision making. To guide AI applications safely, clinicians need some understanding of AI. Numerous commentaries advocate for AI concepts to be taught^1^, such as interpreting AI models and validation processes^2^. However, few structured programs have been implemented, especially on national scales. Pinto Dos Santos et al^3^. surveyed 263 medical students and 71% agreed they needed AI training. Teaching AI to medical audiences requires nuanced design to balance technical and non-technical concepts for learners who typically have a broad range of prior knowledge. We describe our experiences delivering an AI workshop series to three cohorts of medical students and make recommendations for future AI medical education based on this.

## Objectives, timeline, and methodology

**Fig. 1:**
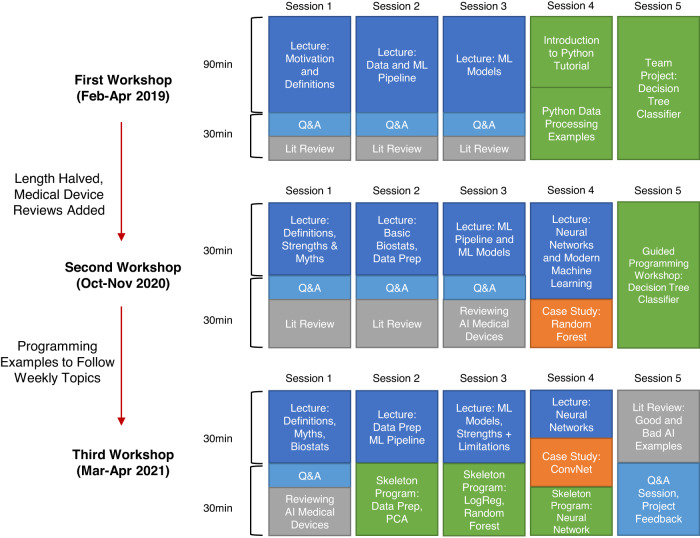
A visualization of the timeline for the three iterations of our workshop. In blue are lecture topics, with light blue dedicated time for interactive question and answer period. In grey are focused brief literature review sessions. In orange are the selected case studies outlining artificial intelligence models or techniques. In green are the guided programming sessions intended to teach how artificial intelligence can solve clinical problems and how to evaluate the model. The content and length with the workshop changed based on evaluation of student needs.

Our five week “Introduction to Medical AI” workshop for medical students was delivered three times between February 2019 and April 2021. A timeline of each workshop summarizing curricular changes is shown in Fig. 1. We had three major learning objectives motivating our curriculum: For learners to understand how data is processed in an AI application, analyze clinical implications of AI literature, and apply opportunities to collaborate with engineers in developing AI.

The first workshop ran from February to April 2019 at the University of British Columbia and all 8 participants provided positive feedback^4^. Due to COVID-19, the second workshop was offered virtually from October to November 2020, with 222 medical students and 3 resident physicians from 8 Canadian medical schools registered. Presentation slides and code were uploaded to an open-access website (http://ubcaimed.github.io). Major feedback from the first iteration included lectures being dense and material being overly theoretical. There was the additional challenge to serve 6 different time zones in Canada. Hence, the second workshop reduced sessions to 1 h each, condensed didactic material, added more case studies, and created template programs to allow participants to complete segments of code with minimal debugging (Box 1). Major feedback from the second iteration included positive reception of programming exercises and requests to demonstrate planning a machine learning project. Hence, in the third workshop which ran from March to April 2021 virtually to 126 medical students we included more interactive programming exercises and a project feedback session to demonstrate critical evaluation of projects using concepts from the workshop.

Box 1 Glossary **Data Analytics:** A field of study in statistics where patterns in data are analyzed, processed, and communicated to identify meaningful patterns in data.

**Data Mining:** The process of identifying and extracting data. In the context of artificial intelligence, this is commonly in large quantities with multiple variables for each sample.

**Debugging:** The processing of finding and resolving unintentional errors in programs.

**Dimensionality Reduction:** The process of transforming data with many individual features to a lesser number of features while retaining significant properties of the original dataset.

**Feature** (in the context of artificial intelligence): A measurable property of a sample. Commonly used interchangeably with “attribute” or “variable”.

**Fourier Transformation:** A technique to convert a periodic signal to individual weighted sinusoids.

**Gradient Activation Map:** A technique for interpreting artificial intelligence models, particularly convolutional neural networks, where the optimization process in final section of the network in analyzed to identify regions of the data or image that have high predictivity.

**Standard Models:** Existing artificial intelligence models that have been previously trained to perform a similar task.

**Testing** (in the context of artificial intelligence): Observing a model performing a task with data it has not been previously exposed to.

**Training** (in the context of artificial intelligence): Exposing a model to data and resulting outcomes for the model to adjust its internal parameters to optimize its ability to perform the task with new data.

**Vector**: An array of data. In machine learning, each element in the array is commonly an unique feature for the sample.

## Curriculum

The most recent curriculum, from April 2021, is summarized in Table 1 and includes the targeted learning objectives for each topic. The workshop was designed for a novice level of technical proficiency, with no mathematics beyond a first-year undergraduate medical course. The curriculum was designed by 6 medical students and 3 instructors with engineering graduate degrees. Engineers proposed AI theory for teaching and medical students filtered for clinically relevant material.

**Table 1:** A summary of concepts taught for each session of the final iteration of the workshop.

Lecture topics (Learning objective achieved)	Case studies and programming examples (Learning objective achieved)
**Introduction and Biostatistics Fundamentals:**	**Case Study: AI Medical Devices**
• AI definitions, misconceptions, strengths (1)	• Problem definition, data collection, model development (1,2)
• Descriptive, inferential, predictive statistics and its limitations (1)	• Regulatory approval (2,3)
• Feature spaces, class balancing, normalization, continuous or discrete data (1)	• Roles of physician, engineers, allied healthcare workers (2,3)
**The Machine Learning Pipeline**	**Programming: Genoa Wine Data**
• Feature spaces, data cleaning (1)	• Exploratory data analysis: looking for outliers, class balance, feature data types (1,2)
• Feature selection methods, feature importances, data augmentation (1)	• Dimensionality reduction with principal component analysis, clustering (1,2)
• Supervised vs. unsupervised learning (1)	
• Training, testing, cross-validation (1)	
**Machine Learning Models**	**Programming: Wisconsin Breast Cancer Data**
• Introducing logistic regression, k-nearest neighbors, random forest, gradient boost(1)	• Training random forest and logistic regression classifier, analyzing performance (1,2)
• Complexity vs. simplicity (1)	• Hyperparameter optimization (1,2)
• Interpreting machine learning models (1,2)	• Extracting of predictive features (1,2)
• Hyperparameters optimization, the “black box effect” and model selection (1)	
**Modern Machine Learning Applications**	**Programming: CIFAR10 Image Data**
• Introducing neural network, convolutional neural networks, weights, neurons, and cost functions (1)	• Normalization of images (1)
• Deep learning and state-of-the-art architectures (1,2)	• Creation of convolutional layers (1)
• How to prepare data and develop neural networks in a clinical environment (1,2,3)	• Training and testing of convolutional neural network (1)
	• Hyperparameter tuning and choosing loss functions (1)
	• Analyzing accuracy and visualization (1)
**Literature Examples and Project Feedback**	**Case Study: AI Literature and Student Projects**
• Summary of objectives and core principles (1,2,3)	• Problem definition, data preprocessing, model selection, validation (1,2,3)
• Resources to learn further technical or clinical artificial intelligence material (3)	• Implications of results, clinical significance, barriers to clinical use (2,3)
• End-to-end description of identification of clinical problem, data preparation, AI model selection, implementation, validation, and deployment (1,2,3)	• Verify proper data science practices, cross-validation, class-balance, proper choice of accuracy metrics (2,3)

Teaching included didactic lectures of core topics, with case studies to demonstrate how to analyze an artificial intelligence (AI) application and skeleton programming examples of an AI solution to a clinical problem where participants are provided with a portion of the code to be completed.

The workshop consisted of lectures, case studies, and guided programming. In the first lecture, we reviewed select data analytics concepts from biostatistics including data visualization, logistic regression, and comparing descriptive versus inferential statistics. Although data analytics is fundamental to AI, we excluded topics such as data mining, significance tests, or interactive visualizations. This was due to time constraints and because several senior students had previous biostatistics training and were keen to cover more unique machine learning topics. The subsequent lectures presented current state-of-the-art methods and discussed AI problem formulation, strengths and limitations of AI models and model validation. Lectures were reinforced with case studies from the literature and from existing AI devices. We emphasized the skills needed to assess model performance and feasibility for a clinical problem, including understanding limitations of current AI devices. For example, we guided students in interpreting a pediatric head trauma guideline by Kupperman et al.^5^, where an AI decision tree algorithm was implemented to determine if computed tomography scanning was beneficial based on a physician’s examination. We highlighted that this is a common example of AI providing predictive analytics for physicians to interpret, rather than a physician replacement.

**Fig. 2:**
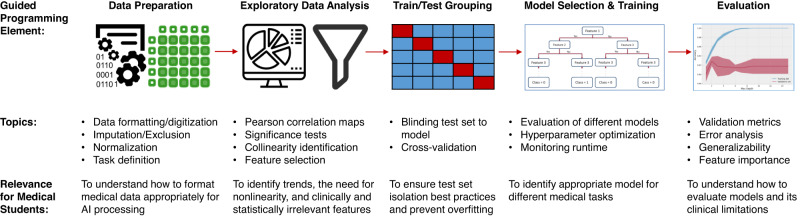
A pipeline of the programming examples developed with specific concepts to be implemented. Programs are introduced during the week of the relevant topic and examples chosen were from published artificial intelligence applications. Programming elements were only included if deemed relevant to provide insight in future clinical practice, such as how to evaluate models to understand if they are ready to be applied in a clinical trial. The examples culminate in a full end-to-end application to classify tumors as benign or malignant based on medical image parameters.

In guided programming examples, available open-source (https://github.com/ubcaimed/ubcaimed.github.io/tree/master/programming_examples), we demonstrated how to conduct exploratory data analysis, dimensionality reduction, loading a standard model, training, and testing. We used Google Colaboratory notebooks (Google LLC, Mountain View, California), which allowed execution of Python code from web browsers. An example of a programming exercise is summarized in Fig. 2. The exercise involved predicting malignant tumors using the Wisconsin Breast Imaging Open Dataset^6^ with a decision tree algorithm.

## Challenges

We identified four main challenges during the training:

1. *Heterogeneity of Prior Knowledge*: Our participants varied in mathematical proficiency. For instance, students with advanced technical backgrounds sought in-depth content such as how to perform Fourier feature transformations. However, it was not feasible to discuss Fourier algorithms to the class as this required advanced signal processing knowledge.
2. *Attendance Attrition*: There was reduced attendance in subsequent sessions, particularly with the online format. A solution could be to track attendance and provide a certificate of completion. Medical schools have been known to provide recognition on student transcripts for extracurricular academic activities, which may incentivize completion.
3. *Curricular Design*: As AI spans numerous subfields, selecting core concepts at an appropriate depth and breadth was challenging. For instance, an important topic is the bench-to-bedside continuum for AI tools. Though we introduced data preprocessing, model construction, and validation, we did not include topics such as mining big data, interactive visualizations, or running an AI clinical trial^7^ in favor of focusing on concepts most unique to AI. Our guiding principle was to train literacy over proficiency. For instance, understanding how a model processes input features is important for interpretability and one method is with gradient activation maps, which visualize which region of data is predictive. However, this requires multivariate calculus and was not feasible to introduce^8^. Developing a shared terminology proved challenging as we struggled to explain how to manipulate data as vectors without mathematical formalism. We noticed different terms shared meanings, such as describing a “feature” as a “variable” or “attribute” in epidemiology.
4. *Knowledge Retention*: It remains to be seen how well participants retain knowledge as there are limited opportunities to apply AI. Medical school curriculums frequently rely on spaced repetition where knowledge is consolidated in practical rotations^9^, which may be applicable to AI education as well.

## Successes

We observed four main successes:

1. *Proficiency was targeted over literacy:* The depth of material was designed without rigorous mathematics, which has been a perceived challenge in launching clinical AI curricula^10^. In programming examples, we used template programs to allow participants to fill in blanks and run software without requiring knowledge of setting up a full programming environment.
2. *Concerns about AI were addressed*: There is a common concern that AI might replace certain clinical duties^3^. To address this, we explained the limitations of AI, including that nearly all AI technologies approved by regulatory bodies require physician supervision^11^. We also emphasized the importance of bias, where algorithms are susceptible to systematic error, especially if the dataset is not diverse^12^. A certain subgroup may hence be modeled incorrectly, leading to inequitable clinical decisions.
3. *Resources were open-access:* We generated publicly available resources, including lecture slides and code. While access to synchronous content was limited due to time zones, the open-source content is a convenient, asynchronous method for learning as not all medical schools have readily available access to AI expertise.
4. *Multidisciplinary Collaboration*: The workshop was a joint venture initiated by medical students to plan curricula alongside engineers. This demonstrated collaborative opportunities and knowledge gaps in both domains for participants to understand potential roles they may contribute to in the future.

## Recommendations

Based on our experience we have four recommendations for others implementing similar courses:

1. *Identify Core AI Competencies*: Defining a list of competencies provides a standardized structure that can be integrated into existing competency-based medical curricula. The workshop currently uses learning objectives levels 2 (understand), 3 (apply), and 4 (analyze) of Bloom’s Taxonomy. Having resources for higher taxonomic levels, such as creation of a project, can further consolidate knowledge. This requires collaboration with clinical experts to identify how AI topics can be applied to the clinical workflow and to prevent teaching redundant topics already included in standard medical curricula.
2. *Create AI Case Studies*: Similar to clinical vignettes, case-based instruction may consolidate abstract concepts by identifying relevance to clinical problems. For example, a study in the workshop analyzed Google’s AI-based diabetic retinopathy detection system^13^ to identify bench-to-bedside challenges such as external validation requirements and regulatory approval pathways.
3. *Use experiential Learning*: Technical skills require deliberate practice and repeated application to master, similar to the learning clinical trainees experience while on rotations. One potential solution is the flipped classroom model, which reported increased knowledge retention in engineering education^14^. In this model, students review theoretical material on their own and class time is used for problem-solving using case studies.
4. *Expand to Multi-Disciplinary Participants*: We envision the implementation of AI involving interaction from various disciplines, including physicians at different levels of training and allied health professionals. As such, curriculum-development in consultation with educators from different faculties may be needed to tailor content for different healthcare domains.

## Conclusions

AI is highly technical, with foundational concepts involving mathematics and computer science. Training medical personnel to understand AI poses unique challenges relating to content selection, clinical relevance, and method used to teach the material. We hope that our insights gained from carrying out AI education workshops may assist future educators of innovative approaches to integrate AI into medical education.

## Data Availability

The Google Colaboratory Python scripts are open-source and available at: https://github.com/ubcaimed/ubcaimed.github.io/tree/master/.
